# Knowledge and attitude regarding Ebola virus disease among medical students of Rawalpindi: A preventable threat not yet confronted

**DOI:** 10.12669/pjms.324.9898

**Published:** 2016

**Authors:** Aliya Hisam, Mariam Nadeem Rana, Mahmood-Ur-Rahman

**Affiliations:** 1 Dr. Aliya Hisam, MBBS, MPH, FCPS. Assistant Professor in Community Medicine Department, Army Medical College, Abid Majeed Road, National University of Medical Sciences (NUMS), Rawalpindi, Pakistan; 2 Mariam Nadeem Rana, MBBS Student, Army Medical College, Rawalpindi, Pakistan; 3 Mahmood-Ur-Rahman, MBBS, DPH, MPH, MSc, FCPS. Professor & Head of Dept. Community Medicine Department, Army Medical College, Rawalpindi, Pakistan

**Keywords:** Attitude, Ebola Virus Disease, Knowledge, Students

## Abstract

**Objective:**

To assess the knowledge and attitude regarding Ebola virus disease (EVD) among medical students of Rawalpindi.

**Methods:**

A descriptive cross sectional study was carried out in a medical college of Rawalpindi from September 2014-November 2014. About 400 students were inducted with 77% (n=308) response rate. After taking informed verbal consent from students and administration, a pre-designed and pre-tested questionnaire was circulated among students of third, fourth and final year MBBS as well as third and fourth year BDS. The data collected was entered and analyzed using SPSS 20.

**Results:**

The response rate was 77% (308/400). About 244 (79.2%) of students had heard about EVD before. One hundred and sixty four (53.2%) of the students correctly identified that no treatment is available for EVD as yet. Also 163 (52.9%) said that no vaccine was available against the virus either. Washing hands every time after touching a patient in clinics/wards was important for 151 (49.0%) while 223 (72.4%) claimed to use proper techniques to dispose off used injections.

**Conclusion:**

Students have basic knowledge regarding EVD. However, there is deficient information regarding the diagnosis and precautionary measures required to control it.

## INTRODUCTION

Ebola Virus Disease (EVD) is an infection caused by a virus of the family *Filoviridae*, genus *Ebolavirus*. Since its discovery in Congo (1976), there have been 27 occurrences in Africa before the current one.^1^ However, the Ebola epidemic of 2014 is the deadliest and longest ever witnessed with 20206 reported cases and 7905 reported deaths according to the WHO fact sheet for 31^st^ December 2014.^2^ It stemmed from Guinea, later spreading on to Liberia, Sierra Leone, Nigeria and Senegal. The disease, once limited to rural areas has now affected the urban population as well.^3^ The present EVD epidemic is caused by the most lethal species of the virus known; the Zaire ebolavirus species with the case fatality rate is as high as 69%. Some believe that the virus was always present in the population while others argue that it has traveled from Central Africa.^4^ All in all, the threat posed by Ebola is so great that on August 8^th^, 2014, WHO declared EVD a ‘Public Health Emergency of International Concern’, urging the entire world to participate in stopping the spread of the viruses.^1^

Ebola viruses are RNA viruses whose genomes encode seven proteins. The glycoproteins are mainly responsible for the pathogenicity of the virus. The body’s monocytes serve as the first line of defence, triggering apoptosis in lymphocytes. The virus infects the endothelial system within three days and soon spreads throughout the body by suppressing the immune system.^1^

The virus is transmitted by eating ‘brushmeat’ or through body fluids of infected people or animals. Caregivers of victims and handlers of the dead infected by the virus are also at particular risk.^5^ Common symptoms of the disease include fever, headache, malaise, vomiting and diarrhea complicated by internal and external bleeding as well as multiple organ failure.^6^ The appearance of a maculopapular rash 5-7 days later which goes on to desquamate is a valuable differential diagnostic criterion.^7^ It is interesting to note that 45.8% of the infections are asymptomatic as reported by Leroy et al.^8^

There is no definitive cure for Ebola as yet. Experimental drugs, for example, Z Mapp are available^5^ with scientific trials underway. Crude treatments include using equine anti-Ebola immunoglobulin with interferon and serum from infected patients.^9^ Similar efforts are in progress to develop a vaccine. A variety of DNA, protein subunit, and several viral vector approaches, both replicating and non-replicating, have been tested as potential vaccines.^10^

EVD is no longer limited to the coast of Africa but has spread around the globe. According to CDC reports, it has tested positive in four patients in the USA.^11^ The deadly virus is also knocking our door with a suspected patient being hospitalized in Karachi recently.^12^ To fight the EVD stigma, we need to recruit and train Popular Opinion Leaders (POL), highly respected members of the community who can rally people to work for a common goal.^13^ Thus, medical students, as doctors of tomorrow have a vital role to play in this battle. Furthermore, the medical staff is most at risk of this contagious disease with patient-to-nurse transmission reported in USA and Spain.^14^ Reasons for the spread of EVD include lack of supplies, healthcare facilities and lack of screening in affected areas^6^, a failure on part of the healthcare community. Hence, it is imperative for them to be prepared for this calamity and contain it before it is too late.

As EVD is not yet prevalent in Pakistan, basic knowledge regarding it is lacking. Thus, we carried out the present study to assess the knowledge and attitude regarding EVD among medical students of Rawalpindi.

## METHODS

It was a descriptive cross sectional study which was carried out in a Medical College, Rawalpindi. Data collection duration was of 3 months from September 2014 to November 2014. Informed verbal consent was taken from the students and administration with confidentiality element maintained. A pre-designed and pre-tested, mixed questionnaire was distributed among students. About 400 students were inducted in the study by non-probability purposive sampling. Students of third, fourth and final year MBBS as well students of third and fourth year BDS participated in the study. Participants were asked to fill the questionnaire and return them back to the community medicine department of Army Medical College. Data was entered and analyzed in Statistical Package for Social Sciences (SPSS) Version 20 for frequencies and percentages calculations. Qualitative variables like knowledge, attitude etc are presented in the form of frequencies and percentages.

## RESULTS

The response rate was 77%. Out of 308 participants, 94 (30.5%) students were male and 214 (69.5%) were female. There were 59 (19.2%) students from 3^rd^ Year MBBS, 89 (28.9%) from 4^th^ Year MBBS, 110 (35.7%) from Final Year MBBS, 33 (10.7%) from 3^rd^ Year BDS and 17 (5.5%) from 4^th^ Year BDS.

A fair number of 244 (79.2%) students had heard about Ebola and 64 (21%) had never heard about it. One hundred and ninety four (63.0%) students were aware EVD was transmitted via body fluids, 196 (63.6%) that it was spread through secretions and 129 (41.9%) that breast milk was responsible for its spread. 162 (52.6%), 172 (55.8%), 122 (39.6%), 128 (41.6%), 146 (47.4%) were aware that EVD was transmitted via saliva, blood, tears, semen and dead body of an infected person respectively.

Fever was the most commonly recognized symptom, identified by 213 (69.2%) students, followed by fatigue identified by 207 (67.2%) students, muscle pain by 190 (61.7%), bleeding by 178 (57.8%), vomiting by 163 (52.9%) and nausea by 153 (49.7%).

A total of 156 (50.6%) students knew that EVD could be diagnosed via ELISA, 124 (40.3%) knew that PCR could be used and 78 (25.3%) were aware that electron microscopy was also an option. Light microscopy was incorrectly identified as a diagnostic technique by 31 (10.1%) of the students and culture by 64 (20.8%). One hundred and sixty four (53.2%) students said that EVD did not have a treatment and 163 (52.9%) said that a vaccine was not available either. Table-I.

**Table-I:** Knowledge and attitude of medical students to EVD (n=308).

Parameters	Variables	Yes n(%)	No n(%)	Do not know n(%)
Heard about Ebola		244(79.2)	64(20.8)	-
Transmission of EVD	Body Fluids	194(63)	34(11)	80(26)
	Secretions	196(63.6)	19(6.2)	93(30.2)
	Breast milk	129(41.9)	46(14.9)	133(43.2)
	Saliva	162(52.6)	34(11)	112(36.4)
	Blood	172(55.8)	32(10.4)	104(33.8)
	Tears	122(39.6)	57(18.5)	129(41.9)
	Semen	128(41.6)	46(14.9)	134(43.5)
	Dead body	146(47.4)	47(15.3)	115(37.3)
Symptoms of EVD (n=308)	Fever	213(69.2)	9(2.9)	86(27.9)
	Fatigue	207(67.2)	13(4.2)	88(28.6)
	Muscle Pain	190(61.7)	19(6.2)	99(32.1)
	Nausea	153(49.7)	36(11.7)	119(38.6)
	Vomiting	163(52.9)	31(10.1)	114(37)
	Bleeding	178(57.8)	30(9.7)	100(32.5)
Diagnosis of EVD (n=308)	ELISA	156(50.7)	22(7.1)	130(42.2)
	PCR	124(40.3)	33(10.7)	151(49)
	Electron Microscopy	78(25.3)	68(22.1)	162(52.6)
	Light Microscopy	31(10.1)	109(35.4)	168(54.6)
	Culture	64(20.8)	80(26)	164(53.2)
Availability of treatment against EVD (n=308)		32(10.4)	164(53.2)	112(36.4)
Availability of vaccine against EVD (n=308)		37(12)	163(52.9)	108(35.1)
Personal protective equipment against EVD (n=308)	Gloves	215(69.8)	18(5.8)	75(24.4)
	Gown	228(74)	6(2)	74(24)
	Face mask	222(72.1)	12(3.9)	74(24)
	Goggles	191(62)	33(10.7)	84(27.3)
	Apron	184(59.7)	36(11.7)	88(28.6)
	Shoe Cover	194(63)	27(8.8)	87(28.2)
	Cap	198(64.3)	30(9.7)	80(26)
Seen/Treated patient with EVD (n=308)		15(4.9)	234(76)	59(19.1)

About 215 (69.8%) students believed that gloves should be worn while handling a patient infected with the Ebola Virus. Students also believed other personal protective equipment could be used which included: gown identified by 228 (74.7%) students, facemask by 222 (72.1%), goggles by 191 (62.0%), apron by 184 (59.7%), shoe cover by 194 (63.0%) and cap by 198 (64.3%) students. Only 51 (16.6%) people believed that there was a chance of recovery from EVD if an infected person reported to a healthcare facility. Table-II.

**Table-II:** Attitude of medical students against EVD (n=308).

	Chance of recovery from EVD on reporting to healthcare facility n(%)	Practice of washing hands after touching patients n(%)	Practice of proper disposal of used injections n(%)
Always	51(16.6)	151(49)	223(72.4)
Usually	83(26.9)	81(26.3)	33(10.7)
Sometimes	64(20.8)	25(8.1)	11(3.6)
Seldom	56(18.2)	11(3.6)	5(1.6)
Never	54(17.6)	40(13)	36(11.7)

A total of 151 (49.0%) said that they practiced proper hand washing techniques after touching patients in their clinical wards/hospitals. A much greater number of 223 (72.4%) said that they dispose off used injections properly after use. Surprisingly fifteen (4.9%) students claimed to have seen/treated a patient suffering from EVD.

## DISCUSSION

Throughout time, there have deadly pathogens wiping off large sections of the human civilization. May it be plague in the Middle Ages or the more recent HIV/AIDS epidemic, these diseases have constantly been intriguing health care providers of their times. EVD is probably the stigma for our era. Even though it not yet prevalent in Pakistan, the high rate of international trafficking means the time is not far when it banks borders with us. This necessitates the need to evaluate the knowledge, attitude and practices of the future doctors of our country to prepare them for this upcoming war.

In Sierra Leone where the EVD epidemic is widespread, 100% of the people had heard of it.^15^ However, in this study, a reasonable percentage of the participants (79.2%) had heard about Ebola. Chippaux et al. reported that all secondary cases of the disease were acquired either due to contact with an infected person or through improper handling of body fluids.^1^ 63.0% of the students correctly identified body fluids as a common source of transmission of the virus. Chippaux et al. went on to highlight that the virus persists in breast milk during convalescence or up to 13 weeks after recovery^1^ which was in the knowledge of 41.9% of our students. The other sources of infection were also correctly identified. If these practices are controlled, the basic reproduction number can fall to 0.3-0.4, offsetting the threat.^16^ The natural reservoir for the Ebola Virus is the African fruit bat. It enters humans when the bats are hunted and may get activated due to an appropriate stimulus resulting in clinical disease.^7^

Moving on, fever (69.2%) and fatigue (67.2%) were the most commonly known symptoms which were in coherence with complaints from confirmed cases of EVD in a study in Uganda.^17^ It is also accompanied by nausea, vomiting, abdominal pain, muscle pain, diarrhea, anorexia, rash and bleeding.^18^ A fairly large number of our students were aware of this.

The classic technique used to diagnose Ebola is electron microscopy. However, as this is time consuming, ELISA is more commonly used^10^ and is also more widely known (50.6%) in our setup than electron microscopy (25.3%). Reverse transcriptase polymerase chain reaction, the most sensitive, specific and rapid test^10^ is in the knowledge of 40.3% of the participants. Ten percent (10.1%) thought light microscopy while 20.8% thought culture, both incorrect techniques, could also be used for diagnosis.

Although a number of recombinant vaccine candidates have been developed in non-human primates, there is so far no effective vaccine available against EVD in humans.^19^ More than half the students (52.9%) were aware of this fact. Almost the same number (53.2%) also knew that there was no specific treatment against it. Treatment is usually just symptomatic.^20^

Personal protective equipment (PPE) e.g. gloves, gown, facemask etc., should essentially be worn while handling a patient infected with EVD. Healthcare workers must follow three basic principles of using PPE. Firstly, it should be donned properly before entering the infected zone, ensuring no part of the skin is exposed. Secondly, it should remain in place during patient care. Lastly, doffing (removing of the PPE) requires a structured procedure, a trained observer and a designated area.^21^ The majority of the participant were of this opinion as well. Perhaps, one of the reasons Ebola is so widespread in Africa is that the under resourced countries there cannot afford such control measures for their health care providers.^6^

EVD is practically an incurable disease with a case fatality rate of almost 100% with the Zaire strain being the most fatal.^6^ However, only 17% of our students were aware of this fact. The best way to control such a fatal disease is to prevent it altogether. Almost half of our students (49%) claimed to be using proper hand washing techniques after touching patients during their clinical training/wards. The importance of this is highlighted by the fact that the Adult Infectious Disease Clinic (AIDC) in a local hospital at Kampala, stationed a nurse outside the clinic who made sure that everyone washed their hands before entering the clinic and after leaving it.^22^ In Sierra Leone, where Ebola is widespread, 66% of the people are reporting a change in attitude by washing hands with water and soap.^15^ Furthermore, it was quite disappointing to note that only 12% of the participants disposed off used injections properly after use. The significance of this practice is supported by the knowledge that the first ever reported fatal case of Ebola in Congo (1976) was transmitted while injecting chloroquine for the treatment of malaria.^23^ Lastly, 5% of the participants claimed to have seen/treated a patient with EVD. This is interesting to note because as yet, not even one case of EVD has been confirmed in Pakistan.

The reason that Ebola is so widespread in Africa today is because they were not prepared for such a calamity. Pakistan is already an under resourced country fighting a war against other communicable diseases such as polio, dengue, measles etc. Further burdening our health system with EVD would be damaging. Thus, by educating our health care providers, policy makers, paramedical staff and community about the preventive measures, we can avoid the preventable grave disease reaching our territory.

## CONCLUSION

Students were having the basic knowledge regarding EVD. However, there was deficient information regarding the diagnosis and precautionary measures required to control it. As such more information about diagnosis and precautionary measures is needed. We should adopt the CDC protocol of ‘Isolate, Identify, Inform,^24^ very firmly. This means that all those arriving from areas where EVD is prevalent should be isolated and screened for the disease. If they are identified for any signs and symptoms of it, the patient and the emergency medical services must be informed of the proper precautionary services.
